# The impact of left-behind experience on urban identity of new-generation migrant workers

**DOI:** 10.1371/journal.pone.0300747

**Published:** 2024-05-02

**Authors:** Fu Linlin, Yihe Chen, Shile Fang, Xinnan Xu, Wenli Kong, Ziqi Liu

**Affiliations:** 1 Institute of Rural Development, Zhejiang Academy of Agricultural Sciences, Hangzhou, Zhejiang, 310021, China; 2 School of Accounting, Zhejiang Gongshang University, Hangzhou, Zhejiang, 310018, China; 3 School of Management, Wenzhou Business College, Wenzhou, Zhejiang, 325035, China; 4 School of Public Affairs, Zhejiang University, Hangzhou, Zhejiang, 310058, China; Zhejiang University, CHINA

## Abstract

We investigate the impact of left-behind experiences on the urban identity of new-generation migrant workers using data from the 2017 China Migrants Dynamic Survey. The results show the following: (1) The left-behind experience is an important factor undermining the urban identity of new-generation migrant workers, and the conclusion remains consistent after robustness checks, such as propensity score matching. (2) Left-behind experiences of both parents away from home had the most significant negative impact on urban identity. (3) The results of the mechanism tests indicate that the left-behind experience exerts an adverse impact on urban identity through the pathways of poorer physical health, more frequent migration, more challenging job search, and stronger dependence on preexisting social networks. The findings of this study also offer policy suggestions for promoting the urban identity of new-generation migrant workers.

## Introduction

Rural migrant workers have long been recognized as essential for long-term economic development and for the growth of allocation efficiency in China. With China’s unique dual economic pattern, there is a huge gap between urban and rural areas in terms of economic strength, public welfare, and social security, spurring waves of the rural labor force to engage in urban non-farm industries since the early 1990s. According to the National Bureau of Statistics, there were 295.6 million migrant workers in China in 2022, accounting for almost 1/3 of the total labor force in China. However, it is difficult for migrant workers to be integrated into urban society due to the *Hukou* system, which strictly separates rural *Hukou* holders from urban public welfare, including child education, medical care and housing. Consequently, most migrant workers tend not to bring their family members together and return to their hometowns after retirement. The phenomenon damages sustainable urbanization and is contradictory to the “Common Prosperity” proposed by the central government.

As stated in the 2022 Migrant Workers Monitoring and Survey Report, which was issued by the National Bureau of Statistics, the percentage of migrant workers who are 40 years old or younger stood at 47.0%. Furthermore, it is noteworthy that the new generation of workers, specifically those born after the 1980s, comprised half of the total migrant workforce. Social integration of the new generation of migrant workers is of great significance to urban stability. As a key research object of migrant workers’ integration into society, this new-generation group exhibits features different from those of the first- and second-generation migrant workers. Unlike their predecessors, they do not share a strong attachment to rural areas while they are simultaneously excluded from urban life and work [1]. Most new-generation migrant workers find themselves in a marginalized and contradictory position, facing a severe identity crisis. This greatly hampers their social integration and hinders the urbanization process. Therefore, it is important to explore the factors that affect the urban identity of new-generation migrant workers and ways to improve their social integration and citizenship, as these endeavors will promote their living standards and employment opportunities and in turn, accelerate urbanization. Stable urbanization also helps absorb the surplus rural labor force, which is conducive to a higher concentration of factors of production, a smaller urban–rural gap, and better socioeconomic development.

This study speaks to a large body of work examining the determinants and consequences of individual identity. Identity, as a key dimension reflecting social integration of the population, profoundly affects individuals’ self-awareness in groups and their sense of belonging in society, which further determines individuals’ ability to engage in productive activities [2]. Drawing on the theoretical framework of identity economics, mutual identity between individuals and their organizations is regarded as the fundamental source of organizational profits and personal income [3]. New-generation migrant workers differ from their first- and second-generation counterparts in career choice, labor intensity, social identity, self-positioning, urban integration, and awareness of equality and rights [4,5]. New-generation migrant workers’ urban identity is defined as their subjective attitude, encompassing self-perception, emotional attachment, and anticipation of the future, shaped by their perception of the differences between urban and rural areas during their interactions with urban residents [6]. New-generation migrant workers face a severe urban identity crisis, mostly due to factors such as self-identity, social integration, and psychological distance of urban residents [7–9].

Some literature focuses on the impact of individual identity on labor market performance. For example, by evaluating immigrants’ adaptation to the customs, rules, and language of the destination society, they reveal their sense of belonging to the ethnic group in the destination society, the host society, and the mainstream culture. They also delve into how this sense of identity affects immigrants’ employment probability, work attitudes, and labor returns in the destination society [10–12]. This work complements these studies by examining the lasting effect of early life experience on individual identity at adulthood.

This study is also related to the growing literature on the early-life experience of being left behind, which has profound and diverse effects on individuals, particularly in terms of cognitive abilities, emotional development, and social skills. Left-behind children, due to a lack of direct education and guidance from their parents for a long period of time, may experience some degree of impediment in their language acquisition, thinking training, and learning ability development. This impediment not only manifests in academic performance, but also potentially affects their future career development and quality of life [13–15]. We add to this line of work by providing empirical evidence that social identity disorder could be an adverse outcome of left behind experience at childhood.

The third type of literature mainly focuses on left behind experience as a main determinant of social integration. For example, [16] analyzed the impact of left-behind experience during childhood on the social integration of the new generation of migrant workers from a life course perspective. The analysis covered three dimensions: economic integration, social adaptation, and psychological integration. The results indicate that left-behind experience has a bidirectional impact on economic integration, a significant impact on psychological integration, but no significant impact on social adaptation. [17] studied the impact of left-behind experience on the urban integration of migrant workers and noted that long-term left-behind experience has a significant negative impact on urban integration, with a greater negative impact on male respondents and the new generation of migrant workers. This study addresses weakened social identity rather than social integration induced by left behind experience. This focus is likely to yield more direct effects, since social identity is the prerequisite for social integration.

The present study offers three major contributions. Firstly, the literature on the impact of left-behind experience on the urban identity of the new generation of migrant workers is relatively limited. By focusing on urban identity (representing the level of psychological integration), we aim to expand this literature. Secondly, we aim to focus more on the long-term dynamic development process of left-behind children, broadening the existing research perspectives and providing new analytical dimensions for understanding the urban integration issues of the new generation of migrant workers. Finally, we further expand the discussion on urban identity and innovatively introduce the perspective of left-behind experience during childhood. Through in-depth examination of the mechanisms between left-behind experience and urban identity, this study reveals the unique impact of different mediating channels on urban identity, enriching our understanding of the formation process of urban identity.

Building on this context, this study empirically investigates the impact of left-behind experiences on the urban identity of new-generation migrant workers using data from the 2017 China Migrants Dynamic Survey (CMDS). The findings were as follows. First, the left-behind experience has a significant negative impact on the urban identity of new-generation migrant workers, and this conclusion remains robust after self-selection bias is addressed. Second, the experience of being left behind by both parents has a greater negative impact on the urban identity of new-generation migrant workers compared to the experience of being left behind by one parent. Third, left-behind experiences impact urban identity through different pathways, such as health status, geographic mobility, job search difficulty, preexisting social networks and income.

## Theoretical analysis and research hypothesis

**Fig 1:**
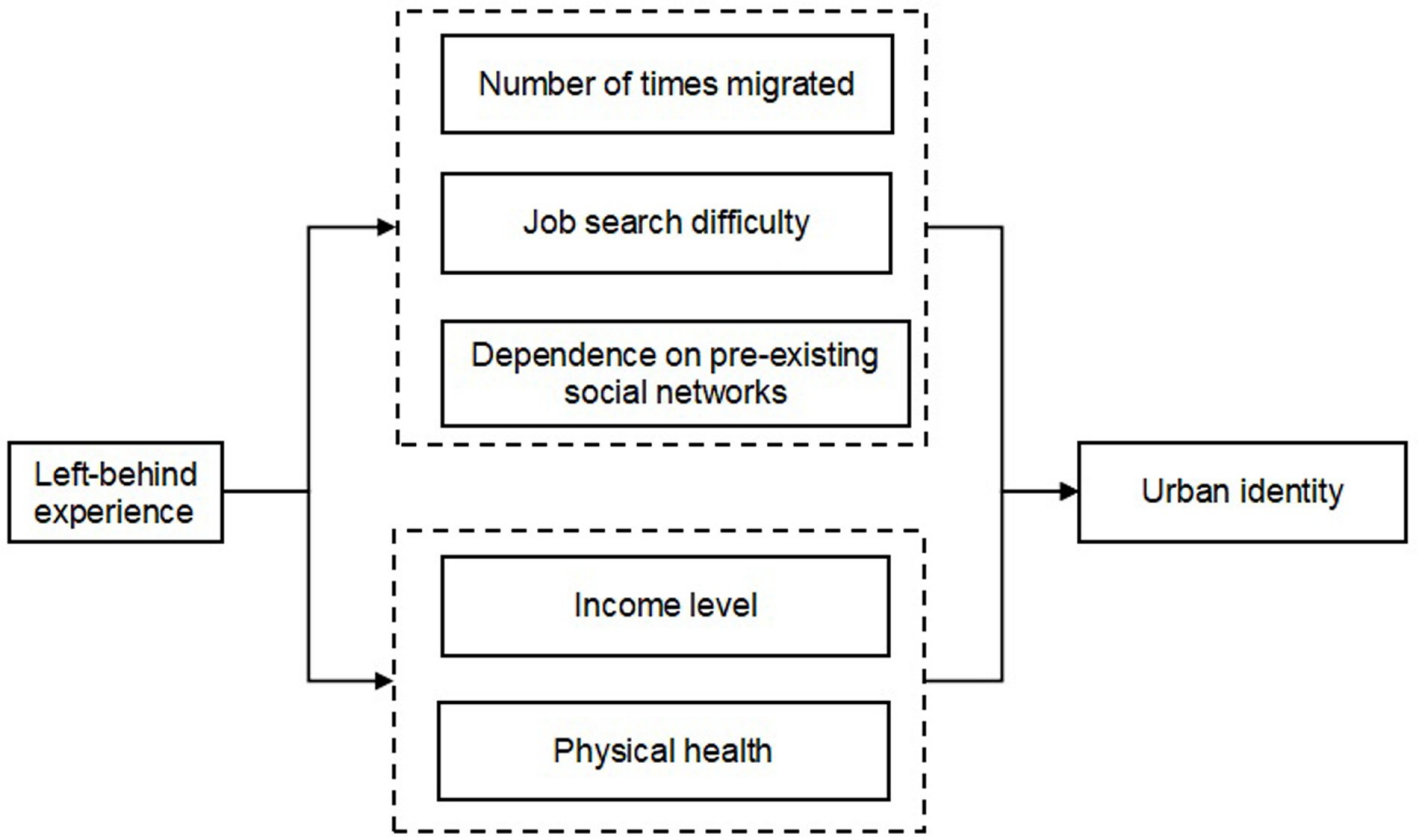
Pathways of left-behind experience affecting urban identity.

Building on the existing literature, we further explore five possible mechanisms to explain how left-behind experience may affect the urban identity of new-generation migrant workers: physical health, social networks, number of times migrated, job search difficulty, and income level as shown in Fig 1.

Different disciplines emphasize the factors that influence migrant workers’ urban integration. Economic theory emphasizes that the role of migrant workers is affected by human capital such as health level [18]. When one or both parents are absent, the children lack appropriate care, resulting in formation of unhealthy eating habits among left-behind children [19], which then lead to malnutrition, increased vulnerability to illness, and other health risks [20,21]. In addition, these children face difficulties in accessing timely and effective medical care once they fall ill or get injured. In terms of mental health, due to the lack of parental care and supervision, rural left-behind children have a greater lack of security and a higher likelihood of depression. Therefore, left-behind children in rural areas are more likely to have mental health problems such as social anxiety and low self-esteem in adulthood [22,23], which may further affect their health status in adulthood. Based on the above analysis, the research hypothesis H_1_ is proposed:

H_1_: Left-behind experience may increase the risk of the new generation of migrant workers falling into poverty in the health dimension, resulting in a lower urban identity.

Sociological theories emphasize the influence of social networks or social capital on the urban integration of migrant workers and hold that, under the condition of incomplete information, “non-market” channels can compensate for the shortcomings of the market to a certain extent [24]. Owing the absence of a father or mother figure, left-behind children are more likely to have frequent contact with individuals from the same village and are more inclined to rely on their existing social networks. Even after working in cities, they continue to maintain frequent contact with their fellow villagers and are reluctant to expand their social networks with locals, resulting in poor social skills [25]. Due to the tendency of the new generation of migrant workers to maintain their original social circles and the difficulty in establishing new social networks with urban residents, there is a lack of trust between groups [26]. This, in turn, hinders their ability to close the psychological distance with urban locals and prevents them from feeling externally recognized for their identity. Consequently, this exacerbates their sense of alienation in the city [27]. Therefore, this article proposes hypothesis H_2._

H_2_: The social relationship network of migrant workers with left-behind experience is highly homogeneous, and they will inevitably maintain their original communication circles, which leads to a lower sense of identity with urban identity.

Psychological capital is a kind of positive psychological state displayed by an individual in the process of growth and development and includes optimism, a sense of responsibility, resilience, and other dimensions [28,29]. The new generation of migrant workers, due to their lack of agricultural experience, have a lower ability to adapt to high-intensity urban work. In the process of integrating into urban society, they tend to be ambivalent between taking root in the city and choosing a job back home. At the same time, they also face psychological deprivation caused by household registration discrimination and ambiguous identity. These factors combined lead to instability in their work and life [30], which leading them to constantly migrate. In addition, due to the parent-child separation caused by the experience of being left behind, the self-centered tendencies of the new generation of migrant workers may be enhanced, and their sense of family responsibility may also be weakened. These factors act together to make it difficult for them to find ideal jobs and living conditions, leading them to be in a state of drifting [31,32]. Therefore, this article posits the following hypothesis H_3._

H_3:_The stronger the geographical mobility of the new generation of migrant workers with left-behind experience, the more adverse it is for them to cultivate urban identity.

Economic integration is the basis for migrant workers’ survival and footholds in the city. According to the search and matching theory, job turnover is an important way for the labor market to achieve efficient allocation of resources. Workers can obtain the most efficient jobs through continuous searching and matching [33]. However, experiences of being left behind may directly affect an individual’s academic performance [34], which, in turn, affects their workplace ability, resulting in relatively low bargaining power in the labor market. In addition, the left-behind experience causes the loss of human capital (education and ability level) of the new generation of migrant workers [35,36], which makes it difficult for them to qualify for their ideal jobs; they thus only engage in jobs with low technical requirements, high intensity [37], poor environmental conditions, and low-income levels. At the same time, they cannot fully enjoy social security and social welfare benefits such as medical care and pension, which lead to lower employment quality, wage income, employment stability, and social security [38]. This reduces their sense of urban identity. Based on this, the following research hypothesis is proposed.

H_4:_ The new generation of migrant workers with the experience of being left-behind face increased difficulty in job search and a lower income level, which in turn reduces their sense of urban identity.

## Data, model, and variables

### Data sources

We used data from the 2017 CMDS, an annual nationwide survey of the migrant population conducted by the National Health Commission since 2009. The CMDS employs a stratified and multistage probability-proportional-to-size sampling method covering 31 provinces (autonomous regions and municipalities directly under the central government) with an annual sample size of nearly 200,000 households. To investigate the impact of left-behind experiences on the urban identity of new-generation migrant workers, we selected individuals born in and after 1980 with rural household registration [37,39]. Relevant data on basic family information, migration scope, and migration tendencies were also retained. Therefore, this study addresses erroneous and missing values for each variable and winsorizes extreme values and outliers for household income before taking the log of the values, thereby mitigating heteroscedasticity. The final dataset comprises 74,158 pieces of data on new-generation migrant workers.

### Model

#### Benchmark regression model

This study established the following model to investigate the impact of new-generation migrant workers’ left-behind experiences on their urban identity:

Iijk=α+β1Lijk+β2'controlijk+ϕj+φk+εijk (1)

In Model (1), *I*_*ijk*_ represents the urban identity of individual i in city j and industry k. *L*_*ijk*_ denotes the left-behind experience, and *control*_*ijk*_ represents the controlled variables of the individual’s personal and family characteristics. *ϕ*_*j*_ refers to city fixed effects, *φ*_*k*_ represents industry fixed effects, and ε_*ijk*_ is the error term. Controlling for city- and industry-level effects eliminates variables that do not change over time, thus minimizing the potential bias caused by omitted variables.

#### Propensity Score Matching (PSM)

Considering that the endogeneity issue—whether new-generation migrant workers have left-behind experience is not randomly assigned—may result in self-selection bias, it is necessary to employ PSM to establish a counterfactual framework to correct this bias. This produces a more accurate net effect of left-behind experiences on urban identity. Drawing on prior relevant literature [40,41], the PSM method divides the sample into treatment and control groups, constructs propensity scores for each individual based on their characteristics, and then matches each individual in the treatment group with another with similar characteristics from the control group. In this study, new-generation migrant workers with left-behind experience were matched with those who had similar characteristics but without such experience to calculate the net effect of the left-behind experience on urban identity. First, appropriate controlled variables were selected, and propensity scores for new-generation migrant workers’ left-behind experiences were estimated using a logistic regression model. The model is expressed as follows:

$$P_{i}(X_{i})=\mathrm{Pr}[L_{i}=1|X_{i}]=E[L_{i}=1|X_{i}]$$ (2)

*X*_*i*_ represents the personal, family, and migration characteristics of new-generation migrant workers. The propensity score for each individual *P*_*i*_(*X*_*i*_) can be calculated using Model (2). Based on the propensity scores, the urban identity inclination of individuals with similar characteristics, but without left-behind experience, can be treated as the potential urban identity inclination of the corresponding left-behind migrant workers. Thus, the Average Treatment Effect on the Treated (ATT) for left-behind experiences on urban identity can be obtained as follows:

$$ATT=E[I_{1i}-I_{0i}|L_{i}=1,X_{i}]=E[I_{1i}-I_{0i}|L_{i}=1,P(X_{i})]$$ (3)

*I*_1*i*_ represents the urban identity inclination of left-behind new-generation migrant workers, and *I*_0*i*_ represents the inclination of their non-left-behind counterparts with similar characteristics. Regarding the specific matching method, this study primarily used a nonparametric estimation, kernel matching. Different weights were assigned to individual samples in the control group. The closer an individual sample is to sample i, the higher the weight assigned and vice versa. This study adopted the *Epanechnikov Kernel* as the kernel function *K*(⋅), with a default bandwidth *h*_*n*_ of 0.06, formulated as follows:

$$ATT=\frac{1}{N^{T}}\sum_{i\in T}\left\{L_{1i}-\frac{\sum_{j\in C}L_{0i}K(\frac{p_{j}-p_{i}}{h_{n}})}{\sum_{j\in C}K(\frac{p_{j}-p_{i}}{h_{n}})}\right\}$$ (4)

where T represents the treatment group and C represents the control group.

### Variables

#### Explained variable: Urban identity

According to the respondents’ answers to the survey question “I am willing to integrate with the locals and become one of them,” this study classified those who answered “completely disagree” and “disagree” as “disagree” (assigned a value of 0) and those who answered “completely agree” and “agree” as “agree” (assigned a value of 1).

#### Key explanatory variable: Left-behind experience

Following the practices of [42], this variable was constructed based on the answers to two survey questions: Question 302 “When did you leave your hometown (county-level) for the first time?” and Question 306 “Did your parents ever work or do business outside of your hometown before your first migration/departure?” Age at first migration was calculated as the difference between the year the respondent left their (county-level) hometown and the year of birth. For respondents who answered “both parents,” “only father,” or “only mother” to Question 306, if their age at the first migration was no older than 18, then the left-behind experience variable was assigned a value of 1; otherwise, if the respondents answered “neither parent” or their age at the first migration was greater than 18, the left-behind experience variable was assigned a value of 0. As the answers “don’t remember” and “migration upon birth” are somewhat vague and only account for 3.06% (2,372 individuals) of the total, these respondents were excluded from the analysis. The results show that the numbers of new-generation migrant workers with and without left-behind experience were 14,377 and 59,781, accounting for 19.4% and 80.6% of the total, respectively.

#### Controlled variables

Drawing on the relevant literature [35,43,44], this study controlled for other variables that may potentially influence urban identity. These included the personal and family characteristics of the new-generation migrant workers. Individual characteristics included gender, age, marital status, years of education, party membership, health records, migration scope, and years of migration; family characteristics included family size, logarithm of family income, and housing. Table 1 provides the definitions and descriptive statistics of the control variables.

**Table 1:** Descriptive statistics.

Variable	Definition	Sample Size	Mean	SD	Min	Max
Urban Identity	Agree = 1; Disagree = 0	74158	0.925	0.263	0.000	1.000
Left-Behind Experience	Yes = 1; No = 0	74158	0.194	0.395	0.000	1.000
Gender	Male = 1; Female = 0	74158	1.524	0.499	0.000	1.000
Age	The respondent’s age in 2017	74158	28.826	4.942	15.000	37.000
Marital Status	Married = 1; Unmarried = 0	74158	1.841	0.686	1.000	6.000
Years of Education	Years of education	74158	10.609	2.864	0.000	19.000
Party Membership	Yes = 1; No = 0	74158	0.032	0.177	0.000	1.000
Health Record	Yes = 1; No = 0	74158	0.261	0.439	0.000	1.000
Migration Scope	Interprovincial = 1; Intra-provincial & intercity = 2; Intra-city & inter-county = 3	74158	1.676	0.756	1.000	3.000
Years of Migration	Difference between 2017 and the year of migration	74158	4.661	4.414	0.000	36.000
Family Size	Number of people in the household	74158	3.078	1.254	1.000	9.000
Logarithm of Family Income	Logarithm of family income	74158	8.685	0.532	7.313	10.309
Housing	Has property = 1; No property = 0	74158	0.228	0.420	0.000	1.000

## Results and discussion

### Benchmark regression results

The benchmark regression results are presented in Table 2. Columns (1) and (2) employ ordinary least squares (OLS) to examine the impact of the left-behind experience on urban identity and cluster the model’s standard errors at the city level by including city and industry fixed effects. To verify the robustness of the results, we report the logit model regression results in Columns (3) and (4).

**Table 2:** Benchmark regression results.

Urban Identity	(1)	(2)	(3)	(4)
Left-Behind Experience	−0.0190***	−0.0108***	−0.2434***	−0.1395***
	(0.0029)	(0.0032)	(0.0362)	(0.0413)
Gender	−0.0134***	−0.0090***	−0.1969***	−0.1439***
	(0.0020)	(0.0023)	(0.0291)	(0.0341)
Age	0.0012***	0.0015***	0.0170***	0.0206***
	(0.0003)	(0.0003)	(0.0036)	(0.0042)
Marital Status	0.0073**	0.0012	0.0975**	0.0134
	(0.0031)	(0.0036)	(0.0434)	(0.0517)
Years of Education	0.0074***	0.0065***	0.1122***	0.1027***
	(0.0003)	(0.0005)	(0.0053)	(0.0067)
Party Membership	−0.0078*	−0.0040	−0.0664	0.1329
	(0.0046)	(0.0050)	(0.0968)	(0.1172)
Health Record	0.0186***	0.0183***	0.3089***	0.3205***
	(0.0020)	(0.0027)	(0.0366)	(0.0454)
Family Size	−0.0044***	−0.0026**	−0.0703***	−0.0449***
	(0.0010)	(0.0013)	(0.0139)	(0.0169)
Logarithm of Family Income	0.0030	0.0084***	0.0620**	0.1319***
	(0.0020)	(0.0028)	(0.0300)	(0.0371)
Housing	0.0362***	0.0177***	0.8347***	0.5315***
	(0.0019)	(0.0028)	(0.0495)	(0.0638)
Migration Scope	0.0216***	0.0191***	0.3466***	0.3124***
	(0.0012)	(0.0020)	(0.0218)	(0.0303)
Years of Migration	0.0026***	0.0017***	0.0397***	0.0256***
	(0.0002)	(0.0003)	(0.0039)	(0.0045)
Intercept Term	0.7434***	0.7032***	−0.2589	−0.3441
	(0.0175)	(0.0249)	(0.2623)	(1.0803)
City Fixed Effects	No	Yes	No	Yes
Industry Fixed Effects	No	Yes	No	Yes
Observations	74158	61816	74158	61816
R-Squared	0.0247	0.0808	0.0510	0.1111

Note: The numbers in parentheses are robust standard errors. *, **, *** represent significance levels of 10%, 5%, 1%, respectively. The same applies below.

As shown in Table 2, the regression results of the OLS and logit models indicate that the impact of left-behind experiences on urban identity is significantly negative at the 1% level of statistical significance. This suggests that new-generation migrant workers with left-behind experiences find it more challenging to maintain an urban identity. The regression results in Column (1) show a larger impact of the left-behind experience on urban identity than those in Column (2). This may be attributed to the omitted variable bias in Column (1) due to the lack of other controlled variables, leading to an exaggerated regression coefficient. In general, the regression results indicate that new-generation migrant workers with left-behind experiences still face negative influences on their urban identity due to their educational level and personal values, even after they migrate to urban areas. Integration into urban life remains a major challenge.

### Robustness check

#### PSM

Table 3 presents the PSM results after kernel matching was applied to the entire sample. As different matching methods may introduce certain biases in the measurement, they may result in varying results, even for the same sample. However, if different matching methods yield similar or consistent results, PSM results are considered robust [45,46]. Therefore, this study reports the results of both one-to-one and one-to-two nearest-neighbor matching and caliper matching. The ATT for each matching method is significantly negative at the 1% level, indicating that the left-behind experience of new-generation migrant workers still has a negative impact on their urban identity, even after the self-selection bias is largely overcome. The estimated coefficients and significance levels of the PSM results were consistent with the benchmark regression results.

**Table 3:** Propensity score matching results.

Matching Method	Treatment Group	Control Group	ATT	Standard Error	T-statistic
Kernel Matching	0.902	0.918	−0.015***	0.003	−5.15
Nearest Neighbor Matching (1:1)	0.902	0.913	−0.108***	0.004	−2.61
Nearest Neighbor Matching (1:2)	0.902	0.913	−0.011***	0.003	−3.12
Caliper Matching	0.902	0.930	−0.028***	0.002	−10.82

To test whether the matching results effectively balanced the data, we conducted a balance test on the PSM results. Table 4 reports the balance test results in which the deviations of most variables significantly decreased after matching, whereas the t-statistics were not significant. Therefore, it can be concluded that the PSM results meet the requirements.

**Table 4:** Balance test results.

Matching Variable	Mean of Treatment Group	Mean of Control Group	Deviation Value	Deviation Value Reduced	T-statistic
Gender	0.503	0.506	−0.8%	88.3%	−0.66
Age	25.902	25.972	−1.4%	98.1%	−1.18
Marital Status	0.594	0.596	−0.5%	98.8%	−0.36
Years of Education	10.423	10.331	3.4%	60%	2.96
Party Membership	0.023	0.025	−0.8%	87.9%	−0.71
Health Record	0.237	0.239	−0.3%	95%	−0.29
Family Size	3.177	3.192	−1.2%	87.6%	−0.94
Logarithm of Family Income	8.746	8.742	0.8%	94.2%	0.67
Housing	0.218	0.218	0.0%	99.3%	0.02
Migration Scope	1.565	1.576	−1.3%	92.7%	−1.18
Years of Migration	5.439	5.246	4.0%	80.1%	3.12

**Fig 2:**
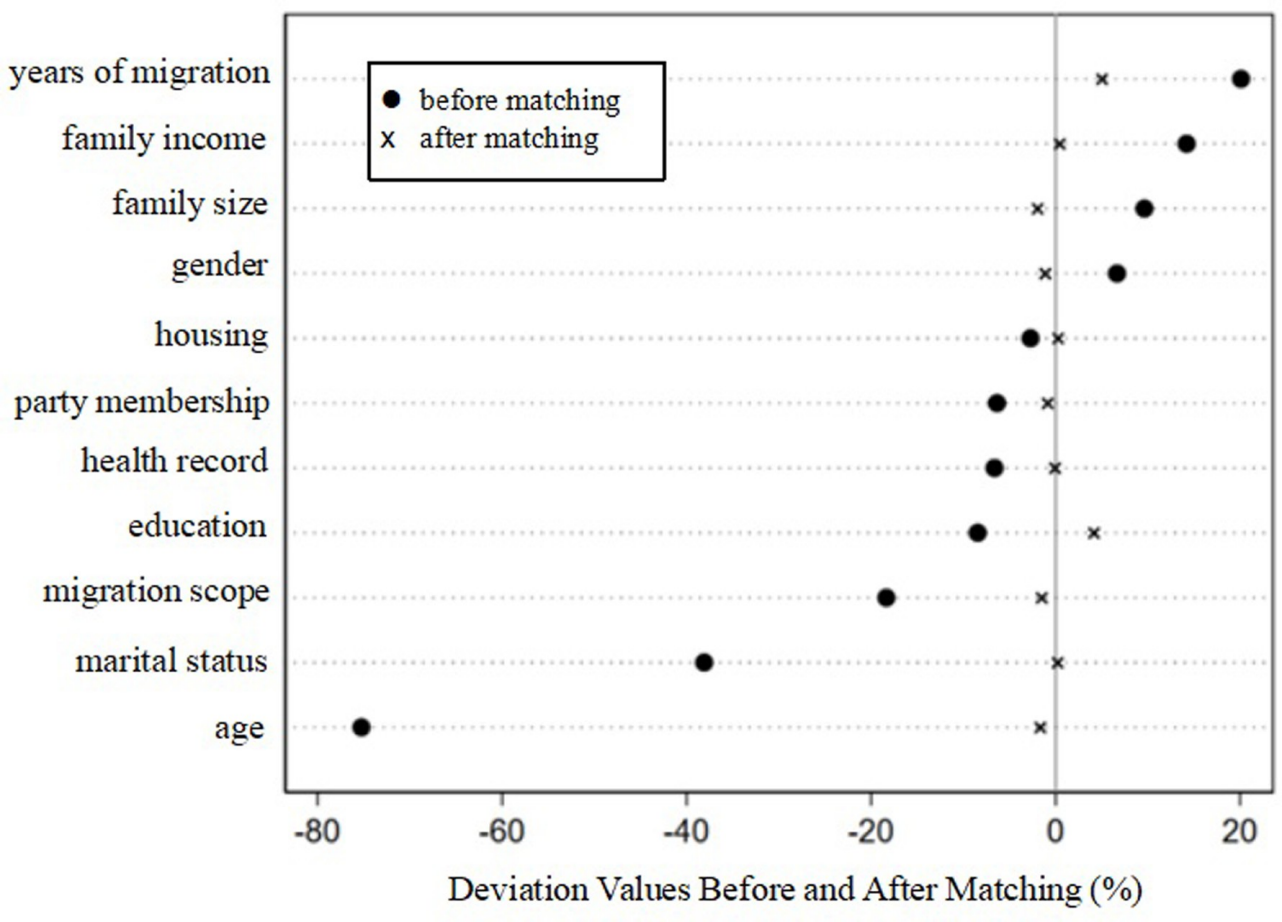
Deviation distribution before and after matching.

**Fig 3:**
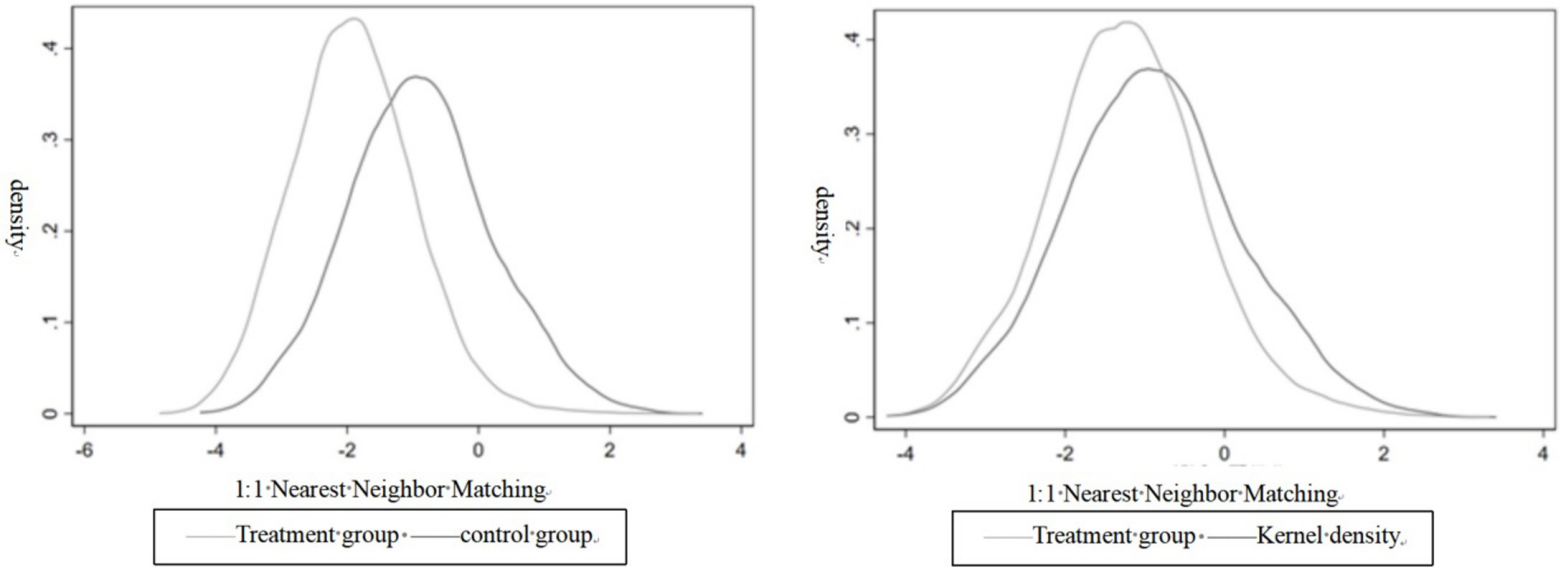
Kernel density plot before and after matching.

Fig 2 shows that the deviations of variables after PSM significantly decreased compared to before matching. Fig 3 provides a kernel density plot for one-to-one nearest-neighbor matching with the probability density distribution of the after-matching samples demonstrating greater convergence. This indicates that the feature variables of the treatment and control groups were largely consistent, effectively reducing self-selection bias.

#### Change the model and remove municipalities directly under the central government

Table 5 presents the results of Poisson pseudo-maximum likelihood (PPML) estimation and regression results after excluding municipalities directly under the central government. To further examine the robustness of the benchmark regression results, we replaced the previous model with the PPML method, which uses iteratively reweighted least squares to calculate the results. As this method does not require assumptions about the distribution of the explained variable, it provides an innovative and robust calculation method for testing the (pseudo-) maximum likelihood estimation.

**Table 5:** Results of PPML estimation and regression results after removing municipalities directly under the central government.

Urban Identity	(1)	(2)	(3)	(4)
	PPML Estimation		Municipalities Directly Under the Central Government Are Removed	
Left-Behind Experience	−0.0208***	−0.0120***	−0.0191***	−0.0099***
	(0.0032)	(0.0036)	(0.0031)	(0.0035)
Personal Characteristics	Yes	Yes	Yes	Yes
Family Characteristics	Yes	Yes	Yes	Yes
City Fixed Effects	No	Yes	No	Yes
Industry Fixed Effects	No	Yes	No	Yes
Observations	74158	61816	64850	54010
RtSquared	0.0009	0.0034	0.0267	0.0859

The regression results in Columns (1) and (2) show that the impact of the left-behind experience on urban identity is significantly negative at the 1% level, which partly ensures the accuracy of the benchmark regression results.

Municipalities directly under the central government are different from ordinary provinces in their economic functions and social positioning [47,48]. They also enjoy relatively abundant resources, large population sizes, great demographic dividends, and policy support while facing issues such as high housing prices and a faster pace of life. This can lead to bias in the benchmark regression results. Therefore, this study reruns the regression after removing four municipalities directly under the central government: Beijing, Shanghai, Chongqing, and Tianjin.

The results in Columns (3) and (4) show that the left-behind experience of new-generation migrant workers has a significant negative effect on their urban identity. However, the regression coefficients are smaller than those of the benchmark regression, indicating that the negative impact on urban identity of municipalities directly under the central government outweighs the positive impact, leading to exaggerated benchmark regression results. Nevertheless, this does not compromise the conclusion of the benchmark regression; the left-behind experience has a significant negative impact on the urban identity of new-generation migrant workers living in ordinary provinces.

### Heterogeneity test

#### Heterogeneity in left-behind experience

New-generation migrant workers are considered to have been left behind if one or both parents were away from home before they came of age. Different types of left-behind experience have varying effects. For instance, [11] discovered that the absences of a father and a mother would have varying impacts on the health of left-behind children. To investigate the impact of different left-behind experiences on the urban identity of new-generation migrant workers, this study divided the left-behind experiences into three groups—both parents away, father away, and mother away—based on the answers to Question 306. Regression analysis was conducted for each group.

Table 6 reports the regression results of different types of left-behind experience. “Both parents away” takes up the majority of the left-behind experience, which has a significant negative impact on urban identity. In contrast, Column (6) shows that the left-behind experience of “only mother away” does not affect the urban identity of new-generation migrant workers. Column (4) demonstrates that the pattern of “only father away” has a certain impact on urban identity, but the corresponding regression coefficient is only significant at approximately the 9% level, close to the nonsignificant threshold of 10%. Therefore, it can be concluded that this pattern also does not significantly affect the urban identity of new-generation migrant workers. Consequently, it can be inferred that the absence of both parents will have a significant impact on the urban identity of new-generation migrant workers while the absence of one parent will not.

**Table 6:** Left-behind experience and urban identity: Heterogeneity in left-behind experience.

Urban Identity	(1)	(2)	(3)	(4)	(5)	(6)
	Both Parents Away		Only Father Away		Only Mother Away	
Left-Behind Experience	−0.0216***	−0.0163***	−0.0275***	−0.0163*	−0.0123	0.0125
	(0.0046)	(0.0049)	(0.0078)	(0.0096)	(0.0184)	(0.0536)
Personal Characteristics	Yes	Yes	Yes	Yes	Yes	Yes
Family Characteristics	Yes	Yes	Yes	Yes	Yes	Yes
City Fixed Effects	No	Yes	No	Yes	No	Yes
Industry Fixed Effects	No	Yes	No	Yes	No	Yes
Observations	18176	14979	6020	4833	916	508
R-Squared	0.0318	0.1257	0.0319	0.2076	0.0330	0.4041

Note:The grouped regression has passed the test of inter-group coefficient differences.

This can be explained by the fact that new-generation migrant workers with the left-behind experience of “both parents away” may have weakened family values as their parents failed to fulfill their expected roles in daily lives. Meanwhile, they are more adaptable to rootless lifestyles than stable lifestyles. Such instability makes it easier for them to migrate between cities, resulting in a weaker urban identity.

#### Heterogeneity in years of migration, migration scope, and gender

The present study grouped new-generation migrant workers based on the following three criteria: more than five years of migration, inter-provincial migration, and gender. Regression analysis was performed for each group.

Table 7 reports the regression results of the three groupings. First, in the case of grouping by years of migration, the left-behind experience of those who migrated to cities more than five years ago does not show a significant impact on their urban identity. However, those with less than five years of migration will suffer from a weaker urban identity due to their left-behind experience. Their urban identity will be enhanced as their social integration increases with the passing of time. Second, a larger migration scope will result in a more significant impact of left-behind experience on urban identity. If new-generation migrant workers have a smaller migration scope, primarily within the same province, their destination and origin cities may have similar economic models, policies, and cultures, which helps them to integrate into the destination city and thus develop a stronger urban identity. Third, female new-generation migrant workers with left-behind experience are less likely to identify with their urban identity than their male counterparts. This may be explained by the fact that female workers are more susceptible to hidden discrimination in their daily lives and work, further diminishing their urban identity.

**Table 7:** Left-behind experience and urban identity: Heterogeneity in years of migration, migration scope, and gender.

Urban Identity	(1)	(2)	(3)	(4)
	Years of Migration ≤ 5 Years		Years of Migration > 5 Years	
Left-Behind Experience	−0.0229***	−0.0123***	−0.0081*	−0.0078
	(0.0037)	(0.0043)	(0.0043)	(0.0049)
Observations	50463	42193	23695	19539
R-Squared	0.0268	0.0937	0.0155	0.1104
Urban Identity	(1)	(2)	(3)	(4)
	Inter-Provincial Migration		Intra-Provincial Migration	
Left-Behind Experience	−0.0275***	−0.0181***	−0.0053	−0.0017
	(0.0044)	(0.0049)	(0.0035)	(0.0041)
Observations	37072	31503	37086	30177
R-Squared	0.0249	0.1050	0.0126	0.0735
Urban Identity	(1)	(2)	(3)	(4)
	Male		Female	
Left-Behind Experience	−0.0183***	−0.0095**	−0.0195***	−0.0132***
	(0.0042)	(0.0046)	(0.0039)	(0.0045)
Observations	35323	33369	38835	28397
R-Squared	0.0200	0.0910	0.0288	0.1084
Personal Characteristics	Yes	Yes	Yes	Yes
Family Characteristics	Yes	Yes	Yes	Yes
City Fixed Effects	No	Yes	No	Yes
Industry Fixed Effects	No	Yes	No	Yes

Note:The grouped regression has passed the test of inter-group coefficient differences.

### Mechanism tests

To investigate the pathways through which left-behind experiences affect the urban identity of new-generation migrant workers, we select health status, geographic mobility, job search difficulty, preexisting social networks, and income as mechanism variables.

#### Health status

We utilize the question "How is your health?" to measure the health status of the new generation of migrant workers. Four options are available for response: healthy, basically healthy, unhealthy but able to care for oneself, and unable to care for oneself. The first two options are classified as “healthy” and assigned a value of 1, while the latter two options are classified as “unhealthy” and assigned a value of 0.

#### Geographic mobility

We measure geographical mobility by the number of cities an individual migrates to. Specifically, this is determined by the response to the questionnaire question, "How many cities have you migrated to in total (including the city you currently reside in)?" Only migrations at or above the county level for the purposes of work and living are included in the count. "Frequent migration" is defined as migrating no less than three times and is assigned a value of 1, while migrating less than three times is considered infrequent and assigned a value of 0.

#### Job search difficulty

The values of the variable are based on the answers to the question, which asks, "What is your major difficulty while living here?" If an individual chooses option, "difficulty in finding a stable job," then the individual sample is assigned a value of 1; otherwise, it is assigned a value of 0.

#### Preexisting social networks

As an indicative variable of preexisting interpersonal networks, its values are obtained from the answers to the question which asks, "Who do you interact with the most in your leisure time here (excluding customers and relatives)?" Among the six options, the first three are all fellow villagers with minor differences in where their households are registered. Therefore, the first three options are assigned a value of one. The other three options are local residents, other migrant workers, and little social interaction, all of which are assigned a value of zero.

#### Income

We measure the income level of the new generation of migrant workers based on the question "What is your last month’s (or current) wage/income?" and take the logarithm of the income variable in the regression analysis.

The regression results presented in Table 8 indicate that the left-behind experience of the new generation of migrant workers has a significant negative impact on their physical health and income levels. Simultaneously, this experience demonstrates a notable positive influence on the geographic mobility, preexisting social networks and job search difficulty of migrant workers. This suggests that migrant workers with left-behind experiences tend to migrate more frequently, face greater challenges in job searching, and have closer interactions with fellow villagers while being reluctant to establish new social networks with urban residents. Consequently, this hinders their sense of urban identity.

**Table 8:** Mechanism test: Left-behind experience and mediating variables.

	(1)	(2)	(3)	(4)	(5)
	Health status	Geographic mobility	Job Search Difficulty	Preexisting social networks	Income
Left-Behind Experience	−0.0017**	0.7559***	0.0185***	0.0171***	−0.0262***
	(0.0007)	(0.0242)	(0.0047)	(0.0052)	(0.0055)
Personal Characteristics	Yes	Yes	Yes	Yes	Yes
Family Characteristics	Yes	Yes	Yes	Yes	Yes
City Fixed Effects	Yes	Yes	Yes	Yes	Yes
Industry Fixed Effects	Yes	Yes	Yes	Yes	Yes
Observations	61816	61816	61816	61816	61186
R-Squared	0.0332	0.1995	0.1397	0.1226	0.4201

## Conclusion

This study empirically investigates the impact of left-behind experiences on the urban identity of new-generation migrant workers using data from the 2017 CMDS. The research findings reveal the following. (1) New-generation migrant workers with left-behind experience are less likely to identify with their urban identity, which remains true after robustness checks such as PSM are employed. (2) The results of heterogeneity tests indicate that the left-behind experience of “both parents away” has the most significant negative impact on urban identity, and longer years of migration and a larger migration scope also prove to have greater adverse impacts on the urban identity of new-generation migrant workers. (3) This study also explored the pathways through which left-behind experiences affect urban identity. Being left behind generally leads to poorer physical health, more frequent migration, more difficult job searches, and a stronger reliance on preexisting interpersonal networks, eventually exerting adverse effects on urban identity.

Based on the aforementioned conclusions, this study proposes a series of specific adjustments to government policies regarding the urbanization of migrant workers.

Firstly, the government must deeply acknowledge the significance of the issue concerning rural left-behind children. These children, often facing numerous challenges due to prolonged separation from their parents, may eventually become the new generation of migrant workers. Therefore, it is the government’s responsibility to strengthen the protection of this demographic. Specifically, policies could be formulated to encourage at least one parent to remain at home and care for the child, ensuring they receive essential companionship and nurturing during critical stages of development. For situations where both parents must work away from home, county and township-level local governments, along with village (community) committees, should play a more prominent role. By establishing effective communication mechanisms, they can guarantee regular contact between parents and left-behind children, fostering a sense of familial warmth and support. Additionally, these grassroots organizations can actively organize and implement various care programs, such as mental health counseling and academic tutoring, to promote the holistic development of left-behind children.

Secondly, the government should take proactive measures in addressing the health and employment challenges faced by migrant workers. As these two factors constitute critical pathways through which the experience of being left-behind influences urban identity, the government should increase its support for the integration of basic medical insurance systems for urban and rural residents. By optimizing the medical insurance system, the economic burden of healthcare for migrant workers can be reduced, thereby improving their health status. Simultaneously, the government should actively promote the enhancement of employment and entrepreneurship policies, providing more job opportunities and entrepreneurial support for the new generation of migrant workers. This not only aids in resolving their economic issues but also elevates their social status and sense of self-identity. Furthermore, the government should continue to emphasize the importance of skills training for workers, offering diversified training programs and learning resources to assist migrant workers in enhancing their comprehensive skill levels and increasing their competitiveness in the urban labor market.

Finally, the government should highlight the significance of establishing local social networks for the new generation of migrant workers. Social networks play a crucial role in individuals’ integration and development within a city. Through establishing connections and interactions with local residents, migrant workers can better understand urban culture and social norms, accelerating their integration into urban life. Additionally, social networks can provide necessary social support and assistance to migrant workers, enabling them to receive timely aid when facing difficulties and challenges. Therefore, the government should implement relevant support programs at the county and township levels, such as community exchange activities and volunteer service projects, to create platforms for migrant workers to interact with local residents. This will contribute to enhancing the sense of belonging and quality of life for migrant workers while preventing excessive reliance on their original social networks.
